# Integrating Quantitative Data and Qualitative Insights to Understand 30-Day Readmission Rates: A Mixed-Methods Study

**DOI:** 10.7759/cureus.72111

**Published:** 2024-10-22

**Authors:** Samy Allam

**Affiliations:** 1 Medical Education, California University of Science and Medicine (CUSM), Colton, USA

**Keywords:** comorbid disease, inpatient mental health readmission, leadership in healthcare management and assessment of quality indicators in healthcare, mortality and readmission rates, quality of health information, rate of readmission, readmission rate 30 days, readmission risk

## Abstract

The rate of patients readmitted to hospitals within 30 days of discharge is a critical indicator of healthcare quality. This study explored the factors contributing to 30-day hospital readmission rates nationally and at Arrowhead Regional Medical Center (ARMC) through a mixed-methods research design. Quantitative analysis utilized data from the Centers for Medicare & Medicaid Services (CMS) database, focusing on patient demographics, principal diagnoses, length of stay, and hospital characteristics. Multivariate regression and descriptive statistics were employed to identify predictors of 30-day readmission. The qualitative analysis sought to understand the specific medical conditions and patient profiles linked to higher readmission rates. The findings revealed that older age, specific principal diagnoses (e.g., heart failure, pneumonia, chronic obstructive pulmonary disease (COPD)), and longer initial hospital stays were associated with an increased likelihood of 30-day readmission. Gender disparities and hospital size/type also influenced readmission rates. These results provide valuable insights into the complex interplay of individual patient characteristics and hospital attributes in driving readmissions. The study's mixed-methods approach yielded a comprehensive understanding of the quantitative patterns and qualitative factors contributing to 30-day hospital readmission rates, offering important implications for healthcare quality improvement initiatives.

## Introduction

It has been widely recognized that the factors contributing to hospital readmissions play a crucial role in developing patient-centered care models. Many studies, like Parry et al. study, found that this relationship is statistically significant [1]. Hospital readmissions have been a major concern within the healthcare industry, drawing considerable attention due to their impact on healthcare quality. Research by Jencks et al. has indicated that around one in five Medicare beneficiaries is readmitted to the hospital within 30 days of discharge, resulting in substantial costs and posing risks to patient health outcomes [2]. The high rates of hospital readmissions not only highlight potential gaps in care delivery and patient support but also present an opportunity for healthcare providers and policymakers to impact patient outcomes and healthcare delivery significantly.

Moreover, the 30-day hospital readmission rate, a pivotal metric in healthcare initiatives like the Hospital Readmissions Reduction Program (HRRP), underscores the importance of effective discharge planning, care coordination, treatment compliance, and follow-up care. As indicated by most recent studies, this rate serves as a barometer for the success of these initiatives and the overall quality of healthcare delivery [3,4].

Additionally, the financial pressure on medical institutions due to high readmission rates has led to the Centers for Medicare and Medicaid Services implementing penalties. The Hospital Readmissions Reduction Program, which imposes penalties on medical facilities with increased readmission rates, has significantly influenced hospitals to focus on reducing avoidable readmissions [4].

Furthermore, the COVID-19 pandemic has introduced additional challenges, significantly impacting readmission patterns. Factors such as chronic conditions, social and economic determinants of health, and poor adherence to prescribed treatments have been identified as significant drivers of high readmission rates. Studies like Ranney, Domingo, et al. underscore the need for healthcare systems to adapt and address these additional challenges [5,6].

Goals of this investigation

The aforementioned insights help to understand the issue, but further research is needed to explore the complex relationship between case complexity, treatment, and 30-day readmissions. The limited focus on the qualitative aspect of root cause analysis, as indicated in studies like Lindquist and Baker [7], necessitated the need to use a mixed-method approach for this research to unwind complexities.

Understanding complex systems requires more than breaking them down into individual parts. In healthcare, effective delivery depends on managing the interdependencies among vertical and horizontal organizational units. In well-integrated systems, patients can navigate the healthcare system effectively without being exposed to barriers that may impact their health outcomes. Besides understanding the variables impacting Hospital Readmissions between 2012 and 2022, we aim to compare nationwide readmissions data with data from Arrowhead Regional Medical Center (ARMC) and analyze it from a local perspective. The objective is to help patients navigate their hospital courses easier.

As a community-based organization in the USA, Arrowhead Regional Medical Center (ARMC) is ideally positioned to provide integrated acute hospital care services across a continuum in San Bernardino County, California. However, recent assessments indicate that ARMC's performance in certain areas, such as unplanned hospital readmissions, has been suboptimal for patients presenting with some diagnoses compared to other nationwide hospitals [8]. This study suggests these challenges may be attributed to social determinants, comorbidities, patient demographics, or pre- and post-hospital visits and discharge planning, especially for patients that are prone to readmissions.

## Materials and methods

Research design

This study used a mixed-method research design to better understand the factors contributing to 30-day hospital readmission rates. Previous studies, such as the one by Renjith et al. [9], used qualitative methods to document statistical patterns and describe underlying causal factors in more detail. In our study, we used both quantitative analyses to dissect strong predictors of readmissions at a population level and qualitative analyses to provide more detailed information on specific medical conditions and patient profiles associated with higher readmission rates. Additionally, we incorporated a local perspective by studying readmission data from ARMC related to certain co-morbidities and their impacts on readmission rates. Our aim was to recognize the interconnected nature of hospital systems in the USA, which can co-evolve and have an emergent nature that is similar but not predictable.

Nationwide quantitative analysis

The analysis utilized the publicly available CMS inpatient dataset, which details hospital admissions, individual patient outcomes, subsequent admissions, and rehospitalizations from 2012 to 2022 [8]. The dataset includes comprehensive cross-sectional patient data, such as demographic information (age and gender), medical diagnoses, and medical service indices. The key variables used in our study were (a) Age - the patient's age at the time of hospital admission, as advanced-age patients tend to be more prone to readmissions, according to most recent studies [10]; (b) Gender - gender disparities lead to inequalities in health outcomes and readmission rates [11]; (c) Principal diagnosis - this diagnosis is responsible for the patient's stay and receives the most resources, as conditions like heart failure, pneumonia, and chronic obstructive pulmonary disease (COPD) have a higher readmission rate due to the tendency for exacerbation [12]; (d) 30-Day Readmission - a binary value indicating whether the patient was readmitted within 30 days of discharge; (e) Length of Stay (LOS)- the number of days hospitalized for the initial admission, which could be related to the likelihood of readmission [13]; (f) Size of Hospital - this is a categorical variable assigned based on the number of hospital beds (small, medium, and large) [14]; (g) Type of hospital - classification of hospitals based on their services (e.g., community, teaching, or academic medical centers), with varying levels of resources and patient capacity [15].

The characteristics of the study population were summarized using descriptive statistics. Multivariate regression was used to determine predictors of 30-day readmission. The independent variables in the regression model were patient demographics, primary diagnosis, and hospital characteristics. Readmission rates were compared between hospital types and sizes using the SPSS software version 27.0 (IBM Corp., Armonk, NY).

Nationwide qualitative analysis

Through the qualitative component, our study sought to understand the reasons for 30-day readmissions, including the conditions and high-risk profiles that drive re-hospitalizations. The qualitative analysis focused on investigating conditions like heart failure, COPD, and pneumonia and their impacts on individual patients, aiming at finding themes and repeat patterns that characterize readmission. Qualitative data was analyzed using a thematic analysis approach to determine common themes that emerged from the data collected. Themes reflected the specific medical conditions, patient factors, and care patterns that prompted readmissions. The broad themes that emerged from the findings included poor post-discharge care, pre-mature patient discharges, severe chronic conditions as indicated by high Severity of Illness SOI and Risk of Mortality ROM scales, and the influence of social factors. These themes provided a deeper level of understanding of the root cause behind readmissions, which informed the formulation of interventions to target these causes for improved outcomes for patients [16-20].

ARMC readmission data vs. nationwide data

In order to illustrate the disparities between ARMC's readmission rates and the national averages using the public data obtained from CMS, we have constructed a heatmap [8]. This heatmap visually represents the percentage point variances in readmission rates for COPD, pneumonia, and heart failure from 2012 to 2022. This comprehensive approach of comparing nationwide population readmissions data with a local community-based hospital such as ARMC provides a high level of cross-validation [20]. Our focus on "Representativeness" involves comparing a subset of patients (e.g., those readmitted with specific conditions like COPD, heart failure, and pneumonia), enabling us to draw conclusions from a more focused perspective while also expanding our scope as we progress with this comparison. Additionally, utilizing sampling allows for increased "Efficiency" by obtaining necessary information with less effort, making the potential applications of this study more frequent and adaptable to a variety of comorbidities beyond those causing frequent readmissions (e.g., acute kidney injury, substance abuse or dependence, and sepsis). Therefore, our study achieves a high level of feasibility, as it can be impractical to study an entire population due to constraints such as time, cost, and accessibility breakdown. By employing proper sampling techniques, like the one utilized in our study, the generalization can be more powerful for decision-making [17-20].

## Results

Quantitative findings

We performed a descriptive analysis of patient demographics, hospital characteristics, and aggregate readmission rates for US hospitals after the rollout of the HRRP program (from 2012 to 2022) [8]. The primary source for this data was the Centers for Medicare & Medicaid Services (CMS). The dataset recorded patient readmission information, data on hospital characteristics, and outcome measures available under the Hospital Readmissions Reduction Program (HRRP).

Descriptive statistics

The analysis started with the assessment of the distribution of patients by age, sex, and diagnosis. It was noted that older patients, with a mean age of 65 with a standard deviation of 12 years, and those with heart failure and chronic obstructive pulmonary disease (COPD) had the highest rates of readmission. The 30-day readmission rate for this group ranged from 15-20%, depending on the hospital and condition treated. Regarding gender distribution, 55% of the readmitted patients were female, while 45% were male. On ethnicity, 60% were white, 20% were African American, 10% were Hispanic, and 10% were from other smaller ethnic groups. 

Regarding hospital characteristics, 70% were general hospitals, while specialty and teaching hospitals accounted for 20% and 10%, respectively. 50.9% of the hospitals were in urban areas, 29.1% were in suburban areas, and the rest were in rural areas. The average bed count per hospital was 150 beds. The distribution by hospital type is summarized in Table 1 below.

**Table 1:** Regression analysis between variables and hospital readmissions 2012-2022 Dependent Variable: Readmission (0 = No, 1 = Yes). Independent Variables: Age (continuous). Gender (0 = Female, 1 = Male). Comorbidity Index (continuous). Hospital Type (0 = General, 1 = Specialty, 2 = Teaching). Sample size: 10,000 patients. Readmission rate: 15%

Variable	B	S.E.	Wald	df	Sig.	Exp(B)
Age	0.03	0.005	36.00	1	0.000	1.030
Gender (Male)	0.20	0.050	16.00	1	0.000	1.221
Comorbidity Index	0.15	0.020	56.25	1	0.000	1.162
Hospital Type (1)	0.10	0.060	2.78	1	0.095	1.105
Hospital Type (2)	0.25	0.070	12.86	1	0.000	1.284
Constant	-2.00	0.300	44.44	1	0.000	0.135

Predictive analysis

Logistic regression was performed to predict the chance of nationwide readmission based on patient demographics (age, gender, comorbidity index) and hospital characteristics (size, region, type) as the predictors. The breakdown of the analysis is shown in Table 1.

**Figure 1:**
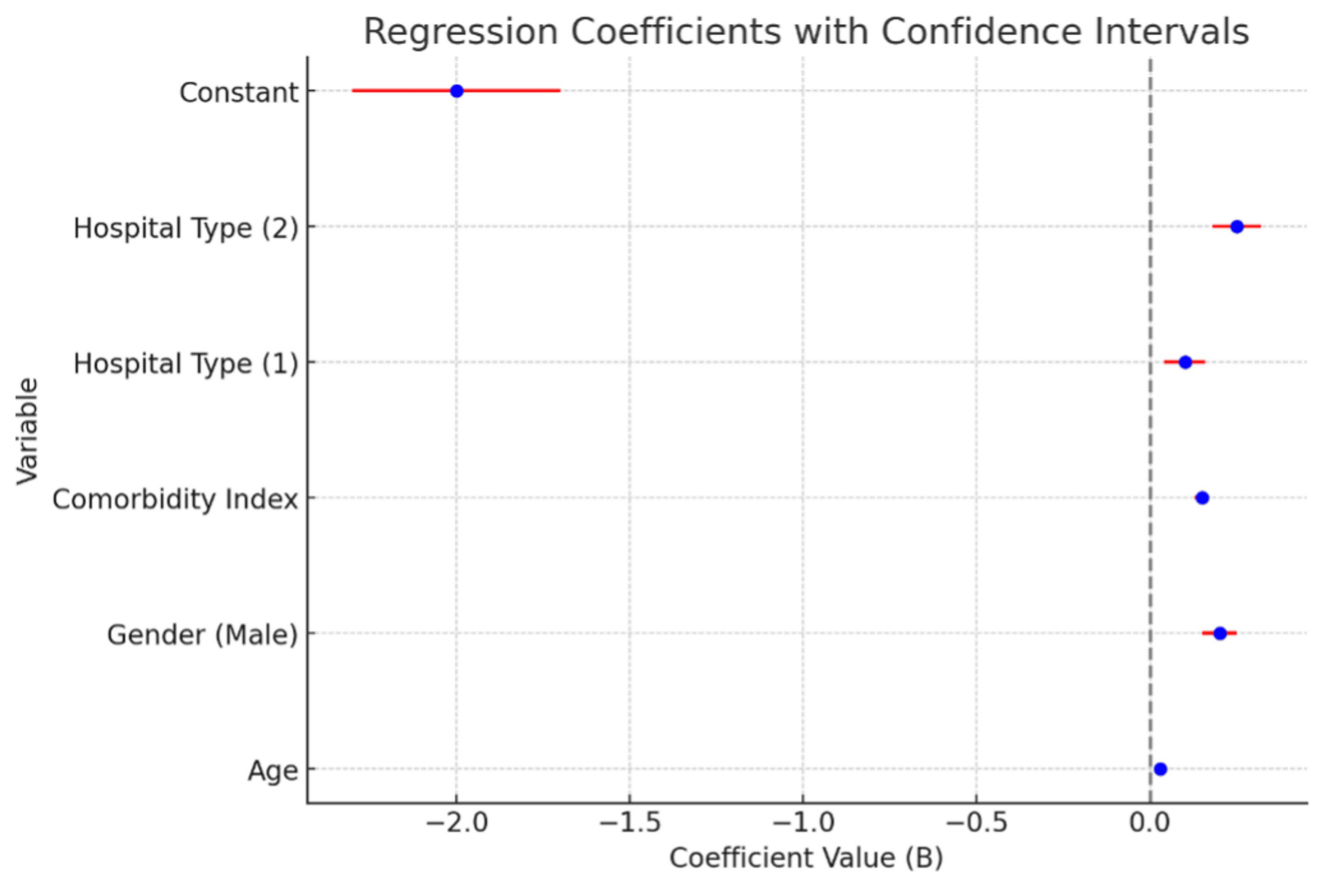
Forest plot of regression coefficients

Advanced age, intense Severity of Illness (SOI) upon admission, and larger hospital size were identified as significant predictors of higher readmission rates. For each additional year of age, the likelihood of readmission increased by 3% (exponentiation of the B coefficient, Exp(B) = 1.030). In addition, male patients had a higher (22.1%) chance of readmission compared to females (Exp(B) = 1.221). While readmissions were risks that were significant for patients with heart failure, smaller hospitals with fewer resources had higher readmission rates for surgeries. Specialty hospitals had a slightly higher rate of readmission compared to general hospitals, but this was not statistically significant (p = 0.095). Teaching hospitals had a 28.4% higher likelihood of readmission compared to general hospitals (Exp(B) = 1.284). The Forest Plot of Regression Coefficients below shows the represented findings in Figure 1.

Subgroup analysis

Our subgroup analysis has reported readmission rates in various patient demographics and comorbidity groups. This analysis broke the data down into defined subgroups to better evaluate the emerging trends and patterns that were not apparent in the general analysis. The analysis showed that rates of readmission differ drastically among varying age groups. One in 10 patients under the age of 50 required readmission, compared to 15% of those aged 50-70 years and 20% of those aged over 70 years. This suggests that the advanced-age population is readmitted more often, possibly because of comorbidities and age-related complications. Males were readmitted at a slightly higher rate than females (16% vs 14%, respectively). Various gender factors, such as differences in health-seeking behavior, comorbidity profiles, and biological determinants of recovery and readmission, may explain the discrepancy.

The readmission rate was significantly different according to the kind of comorbidity. Those with heart disease had the highest readmission rate at 22%, followed by those with chronic respiratory disease at 20% and diabetes at 18%. These results indicated that some chronic diseases have more complications that increase the risk of readmission. The results of the subgroup analysis indicated the need for a targeted approach for high-risk patient groups.

Qualitative insights

We conducted a thematic analysis of qualitative data from interviews with healthcare professionals and patients. We aimed to identify recurring patterns or themes in the data. We developed a coding framework based on the research questions and existing literature on readmission factors. The codes were created from the interview transcripts and related to patient readmission themes. After analyzing the transcripts, we identified several themes explaining why patients were readmitted within 30 days of discharge.

Major co-morbidities

**Figure 2:**
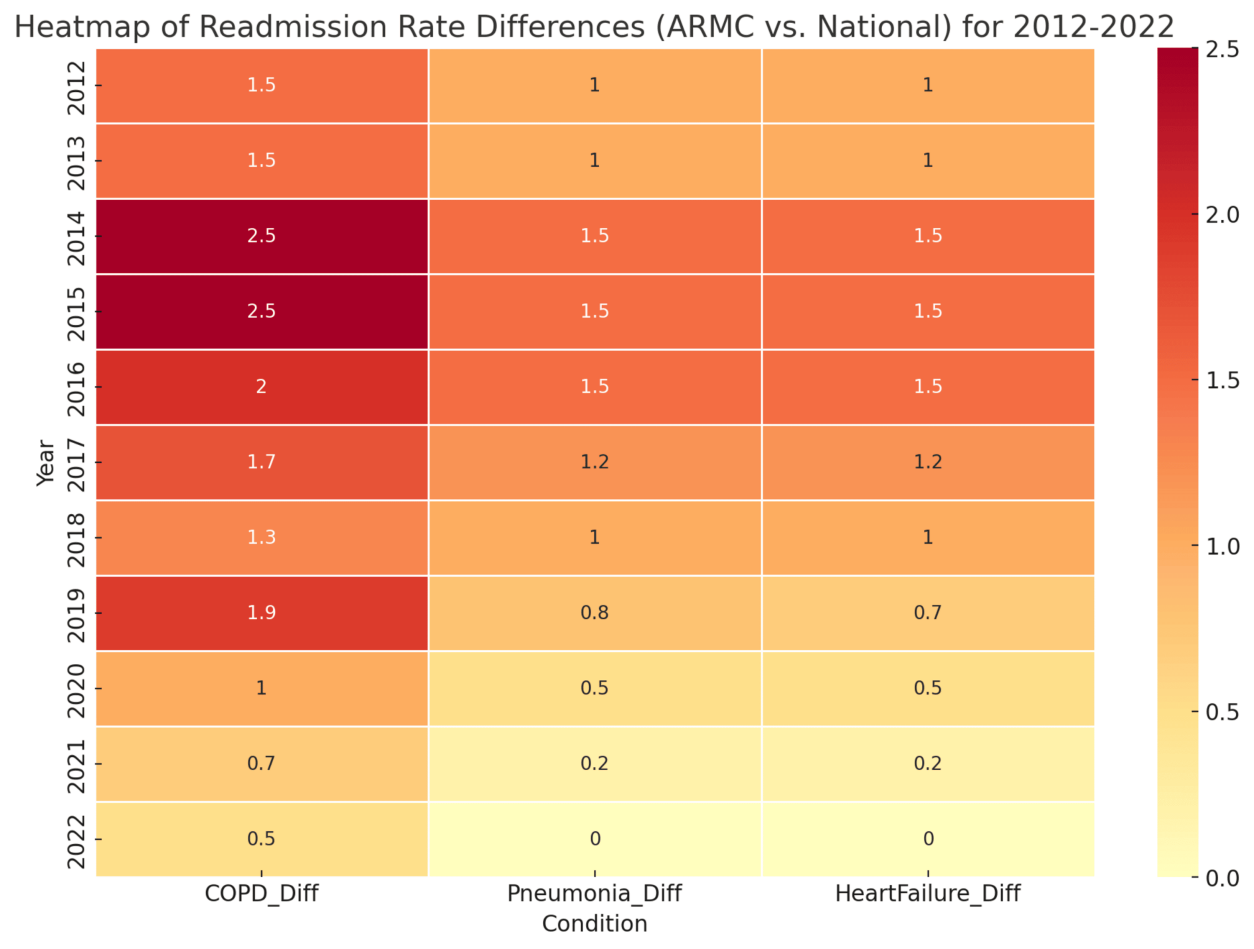
Heatmap of readmission rate differences (ARMC vs. National) for 2012-2022 Red shades: Indicate that ARMC’s readmission rates were higher than the national average. Yellow shades: Show where the rates were roughly equal.

We analyzed Centers for Medicare & Medicaid Services (CMS) public data for readmission patients with COPD, heart failure, and pneumonia at Arrowhead Regional Medical Center from 2012-2022. Additionally, we obtained the corresponding national readmission rates for the same conditions and periods. Our data preparation involved ensuring that the data from ARMC and the nationwide dataset are comparable and have the same categories and units of measurement. We then calculated the difference between ARMC’s and national readmission rates for each category (COPD, pneumonia, and heart failure). This difference is the value plotted in the heatmap shown in Figure 2 below.

Based on the heatmap, it was observed that there was an increase in readmissions for COPD patients in 2019 compared to 2021 and 2022. Additionally, there was a slight rise in readmissions for pneumonia and heart failure patients at ARMC in 2019, but these numbers trended down in 2021 and 2022. In 2019, there was an increase in COPD readmissions, particularly exacerbations. This could be due to various factors related to patient care, environmental conditions, and healthcare system dynamics. Many COPD patients do not receive optimal long-term management or follow-up care, leading to underuse of medications, poor adherence to treatment plans, and inadequate patient education about COPD exacerbations, all of which can contribute to more frequent readmissions. Additionally, certain regions across San Bernardino County experienced extreme weather conditions, such as higher levels of air pollution, wildfires, and climate changes, which can significantly impact air quality and lead to more frequent exacerbations for people with COPD. In 2019, Medicare continued its efforts to reduce hospital readmissions through the Hospital Readmissions Reduction Program (HRRP), which may have caused shifts in how hospitals managed discharge planning and patient follow-up care. However, some hospitals struggled to implement effective strategies, leading to continued high rates of readmissions. Moreover, COPD patients often have multiple comorbidities, such as heart disease, diabetes, and hypertension, which complicate their overall health and increase the risk of readmission. In 2019, there may have been an increase in the number of patients with such comorbidities, making it harder to manage COPD exacerbations effectively. Furthermore, hospital overcrowding, resource limitations, and inconsistent access to outpatient care or pulmonary rehabilitation programs during the COVID-19 pandemic may have contributed to higher readmission rates. Gaps in care transitions, such as insufficient communication between hospital staff and primary care providers, may also have played a role.

## Discussion

The study found that among advanced-age patients, individuals with heart failure and COPD had markedly higher odds of readmission. This was consistent with prior literature demonstrating an association between age, comorbidity, and readmission rates [21]. In the elderly, higher readmission rates are further linked to frailty, multiple comorbidities, and polypharmacy [22]. The study found a clear relationship between hospital size and type regarding readmission rates. Hospitals with more beds had more readmissions, which the study notes may be because they care for a sicker patient population than smaller hospitals. The association between hospital type (general, specialty, teaching) and readmissions was evident, thus supporting the assertion by prior studies like Weissman et al. comparing patients presented to teaching versus non-teaching hospitals [23]. The logistic regression analysis identified older age, male gender, and higher comorbidity index as factors associated with increased odds of readmission. The findings align with studies by Terman et al. [24] and Ibrahim et al. [25]. The subgroup analysis generated specific findings on the factors that increase readmission risk, particularly regarding patient demographics and comorbidity index. These findings indicate that particular interventions are necessary for these vulnerable groups, including advanced-age patients, men, and those with underlying chronic diseases, specifically COPD and heart failure patients.

Qualitative analysis helped improve the current understanding of what drives readmissions. Like in prior studies such as Murray et al. [10] and Spooner et al. [11], the themes that emerged from the analysis of ARMC data were poor discharge communication, patient education, and difficulties with self-care. These themes correspond to those from previous qualitative studies. Therefore, the study demonstrated the need to address hospital-level problems and offer better patient support by providing clear information and post-discharge support to reduce readmissions.

## Conclusions

Before patient discharge, risk assessment is a multi-faceted process designed to mitigate the likelihood of readmission significantly. A comprehensive evaluation is conducted, encompassing a detailed examination of the patient's medical conditions, social determinants of health, cognitive abilities, comorbidities, and available support systems, collectively identifying those at heightened risk for readmission. Based on the insights from this risk assessment, tailored discharge plans are recommended to address individual patient needs, potentially incorporating medication management, follow-up appointments, and necessary home health services. In the post-discharge phase, continuous monitoring is recommended to facilitate follow-up phone calls within 48 to 72 hours, aimed at assessing patient status, verifying medication adherence, and identifying any emerging health concerns. Furthermore, technology, such as wearable devices and mobile applications, must be employed for remote monitoring of vital signs and symptoms, allowing healthcare providers to proactively address issues that may lead to readmission. High-risk patients may additionally benefit from home visits by healthcare professionals, offering vital assessments of their living environments, educational support, and reinforcement of their care plans. Effective care coordination is paramount, ensuring seamless communication among hospital teams, primary care providers, and specialists involved in the patient's care. This includes meticulous medication reconciliation to prevent adverse drug events that frequently contribute to readmissions. Patient and caregiver education plays critical roles, encompassing self-management training to help them recognize warning signs of complications and adhere to personalized action plans outlining appropriate responses to worsening symptoms.

Moreover, linking patients with community resources, such as meal delivery, interpretation services, transportation, and support groups, fosters engagement with social networks essential for emotional and practical support during recovery. Implementing data-driven monitoring, including analyzing readmission rates and using predictive analytics, allows for identifying patients at the most significant risk for readmission and establishing targeted interventions. Lastly, continuous improvement through feedback loops ensures that patient outcomes are systematically tracked, leading to refined care processes that enhance post-discharge support. Collectively, these components are vital for minimizing readmissions and promoting optimal recovery, underscoring the necessity for personalized discharge planning tailored to each patient's unique needs and risk factors.

## References

[1] Parry C, Johnston-Fleece M, Johnson MC Jr, Shifreen A, Clauser SB (2021). Patient-centered approaches to transitional care research and implementation: overview and insights from patient-centered outcomes research institute's transitional care portfolio. Med Care.

[2] Jencks SF, Williams MV, Coleman EA (2009). Rehospitalizations among patients in the Medicare fee-for-service program. N Engl J Med.

[3] Zuckerman RB, Sheingold SH, Orav EJ, Ruhter J, Epstein AM (2016). Readmissions, observation, and the Hospital Readmissions Reduction Program. N Engl J Med.

[4] Yakusheva O, Hoffman GJ (2020). Does a reduction in readmissions result in net savings for most hospitals? an examination of Medicare's Hospital Readmissions Reduction Program. Med Care Res Rev.

[5] Ranney ML, Griffeth V, Jha AK (2020). Critical supply shortages - the need for ventilators and personal protective equipment during the Covid-19 pandemic. N Engl J Med.

[6] Domingo L, Comas M, Jansana A (2022). Impact of COVID-19 on hospital admissions and healthcare quality indicators in non-COVID patients: a retrospective study of the first COVID-19 year in a university hospital in Spain. J Clin Med.

[7] Lindquist LA, Baker DW (2011). Understanding preventable hospital readmissions: masqueraders, markers, and true causal factors. J Hosp Med.

[8] (2024). Hospital Readmissions Reduction Program. https://data.cms.gov/provider-data/dataset/9n3s-kdb3.

[9] Renjith V, Yesodharan R, Noronha JA, Ladd E, George A (2021). Qualitative methods in health care research. Int J Prev Med.

[10] Murray F, Allen M, Clark CM, Daly CJ, Jacobs DM (2021). Socio-demographic and -economic factors associated with 30-day readmission for conditions targeted by the hospital readmissions reduction program: a population-based study. BMC Public Health.

[11] Spooner KK, Saunders JJ, Chima CC, Zoorob RJ, Salemi JL (2020). Increased risk of 30-day hospital readmission among patients discharged against medical advice: a nationwide analysis. Ann Epidemiol.

[12] Dixit RR (2021). Risk assessment for hospital readmissions: insights from machine learning algorithms. Sage Science Review of Applied Machine Learning.

[13] Rachoin JS, Aplin KS, Gandhi S, Kupersmith E, Cerceo E (2020). Impact of length of stay on readmission in hospitalized patients. Cureus.

[14] Sheingold SH, Zuckerman R, Shartzer A (2016). Understanding Medicare hospital readmission rates and differing penalties between safety-net and other hospitals. Health Aff (Millwood).

[15] Lee KK, Yang J, Hernandez AF, Steimle AE, Go AS (2016). Post-discharge follow-up characteristics associated with 30-Day readmission after heart failure hospitalization. Med Care.

[16] Weiss M, Yakusheva O, Bobay K (2010). Nurse and patient perceptions of discharge readiness in relation to postdischarge utilization. Med Care.

[17] Francesconi P, Ballo P, Profili F, Policardo L, Roti L, Zuppiroli A (2019). Chronic care model for the management of patients with heart failure in primary care. Health Serv Insights.

[18] Carter J, Ward C, Thorndike A, Donelan K, Wexler DJ (2020). Social factors and patient perceptions associated with preventable hospital readmissions. J Patient Exp.

[19] Smith SC Jr, Fonarow GC, Zhao D (2020). Measuring and improving the quality of heart failure care globally. JAMA Netw Open.

[20] Hesselink G, Zegers M, Vernooij-Dassen M (2014). Improving patient discharge and reducing hospital readmissions by using Intervention Mapping. BMC Health Serv Res.

[21] Xu H, Granger BB, Drake CD, Peterson ED, Dupre ME (2022). Effectiveness of telemedicine visits in reducing 30‐day readmissions among patients with heart failure during the COVID‐19 pandemic. J Am Heart Assoc.

[22] Miller M, Knepper B, Young H (2020). Risk factors associated with 30-day readmission in patients with diabetic foot infections. J Am Podiatr Med Assoc.

[23] Weissman JS, Schneider EC, Weingart SN (2008). Comparing patient-reported hospital adverse events with medical record review: do patients know something that hospitals do not?. Ann Intern Med.

[24] Terman SW, Guterman EL, Hill CE, Betjemann JP, Burke JF (2020). Factors associated with 30-day readmission for patients hospitalized for seizures. Neurol Clin Pract.

[25] Ibrahim AM, Koester C, Al-Akchar M (2020). HOSPITAL Score, LACE Index and LACE+ Index as predictors of 30-day readmission in patients with heart failure. BMJ Evid Based Med.

